# Supplementary material for: Self-DNA Inhibition in Drosophila melanogaster Development: Metabolomic Evidence of the Molecular Determinants
Source: Biology (Basel). 2023 Oct 27;12(11):1378. doi: 10.3390/biology12111378 (PMC10669329; doi:10.3390/biology12111378)
Supplement: Supplementary file 1 [file biology-12-01378-s001.zip › biology-2666941-supplementary.pdf]

Article

Self-DNA inhibition in Drosophila melanogaster development: metabolomic evidence of the molecular determinants

SUPPLEMENTARY MATERIAL

Figure S1. ^{1}H NMR spectra in triplicates of the *Drosophila* larvae apolar extracts registered in $CDCl_3$ at 600 MHz.

Figure S2. Representative LC-MS chromatograms of polar extracts for each treatment (Sa and Sb) and control (Ca and Cb).

Figure S3. Molecular networking of all *D. melanogaster* polar extracts.

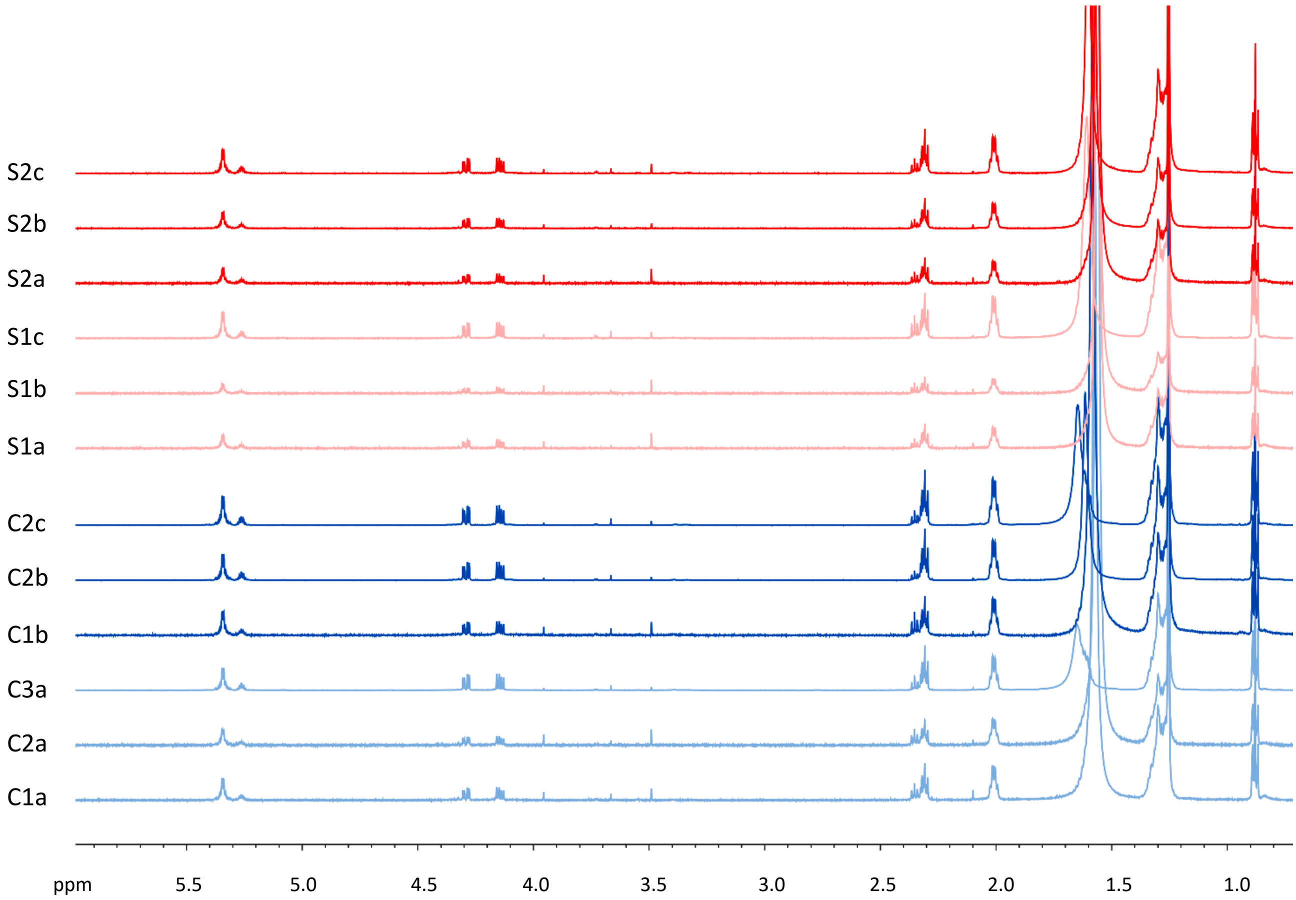

Figure S1. ^{1}H NMR spectra in triplicates of the *Drosophila* larvae apolar extracts registered in $CDCl_3$ at 600 MHz. In red self-DNA at longer exposition times (S1b, S2b and S3b); in light red self-DNA at exposition times (S1a, S2a and S3a). In blue control samples at longer exposition times (C1b, C2b and C3b); in light blue control samples at shorter exposition times (C1a, C2a and C3a).

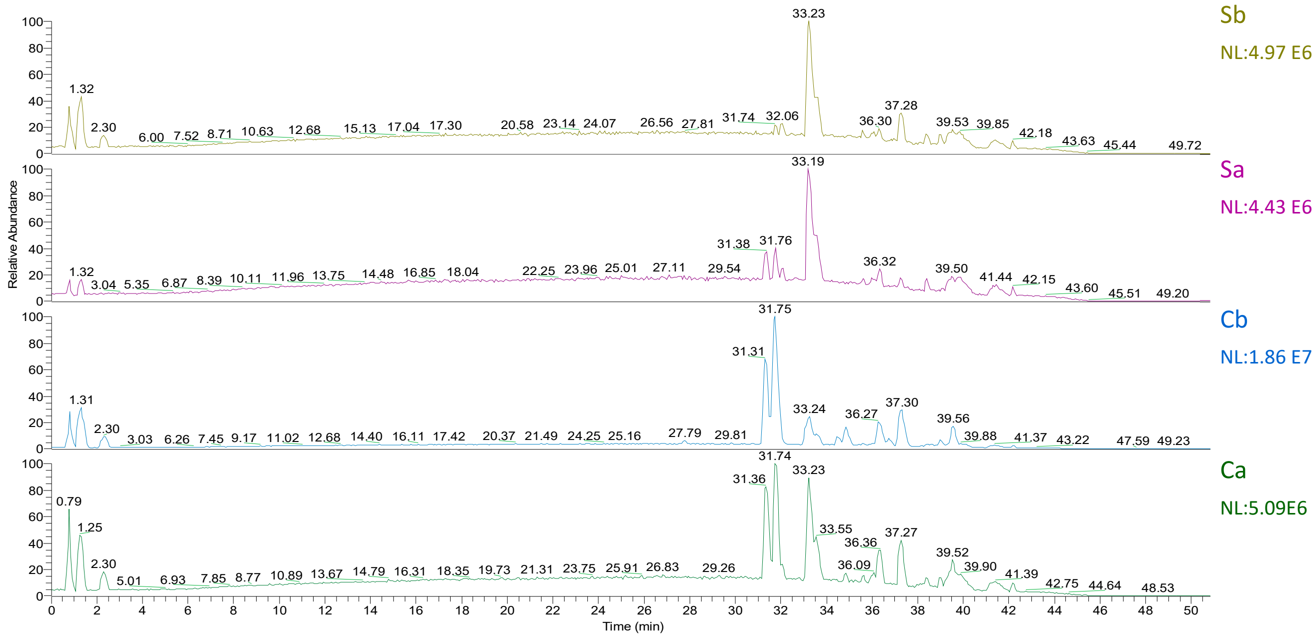

Figure S2. Representative LC-MS chromatograms of polar extracts for each treatment (Sa and Sb) and control (Ca and Cb).

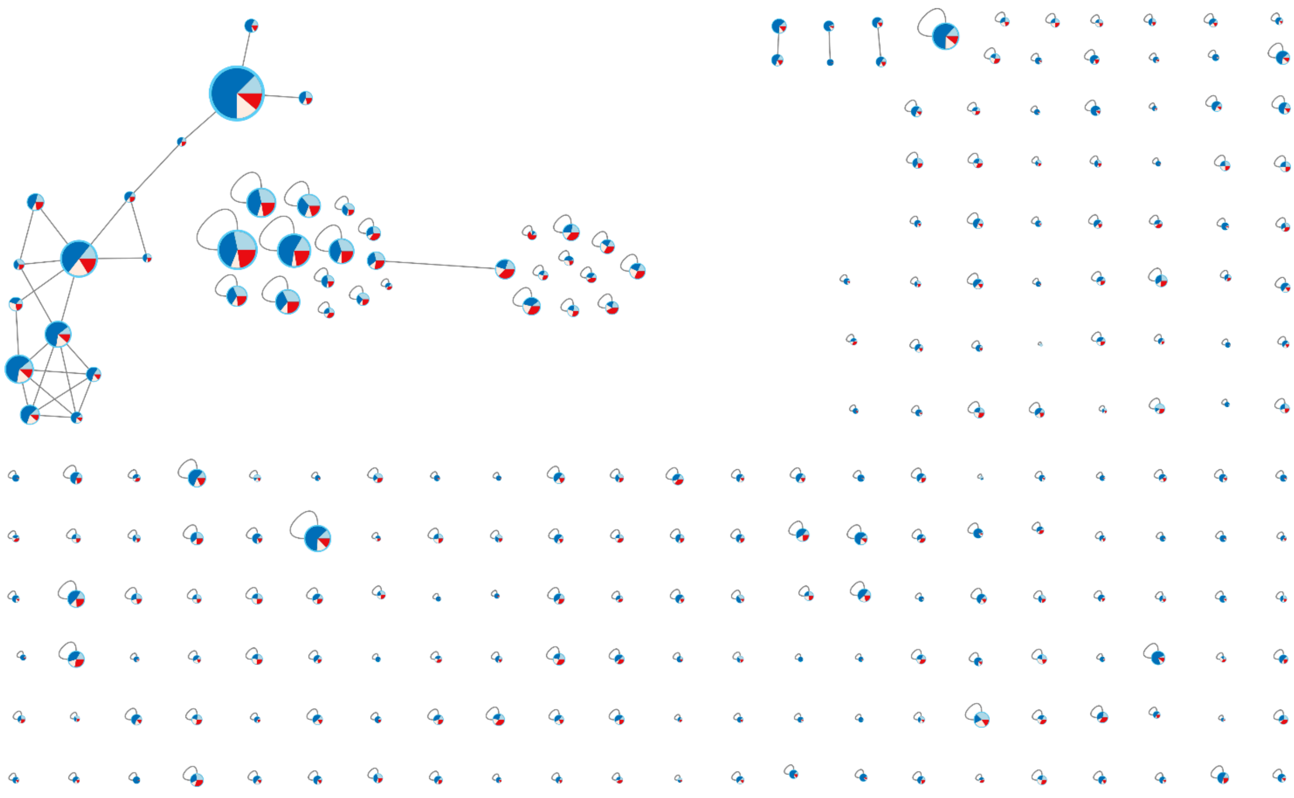

Figure S3. Molecular networking of all *D. melanogaster* polar extracts. Each node is represented as a color-coded pie-chart. Red slices refer to the sum of all samples exposed to self-DNA for 96h (S1b, S2b and S3b); light red slice to all samples exposed to self-DNA for 72h (S1a, S2a and S3a); blue slices to control samples at longer exposition times (C1b, C2b and C3b); light blue slices to control samples at shorter exposition times (C1a, C2a and C3a).
